# Broad Spectrum Antiviral Properties of Cardiotonic Steroids Used as Potential Therapeutics for Emerging Coronavirus Infections

**DOI:** 10.3390/pharmaceutics13111839

**Published:** 2021-11-02

**Authors:** Young-Hee Jin, Sangeun Jeon, Jihye Lee, Seungtaek Kim, Min Seong Jang, Chul Min Park, Jong Hwan Song, Hyoung Rae Kim, Sunoh Kwon

**Affiliations:** 1 KM Application Center, Korea Institute of Oriental Medicine, Daegu 41062, Korea; 2 Center for Convergent Research of Emerging Virus Infection, Korea Research Institute of Chemical Technology, Daejeon 34114, Korea; minseongjang@kitox.re.kr (M.S.J.); parkcm@krict.re.kr (C.M.P.); jhsong@krict.re.kr (J.H.S.); hyungrk@krict.re.kr (H.R.K.); 3 Zoonotic Virus Laboratory, Institut Pasteur Korea, Seongnam 13488, Korea; sangeun.jeon@ip-korea.org (S.J.); jihye.lee_01@ip-korea.org (J.L.); seungtaek.kim@ip-korea.org (S.K.); 4 Department of Non-Clinical Studies, Korea Institute of Toxicology, Daejeon 34114, Korea; 5 KM Convergence Research Division, Korea Institute of Oriental Medicine, Daejeon 34054, Korea

**Keywords:** cardiotonic steroid, coronavirus, MERS, COVID-19, antiviral, pharmacokinetics

## Abstract

Cardiotonic steroids are steroid-like natural compounds known to inhibit Na^+^/K^+^-ATPase pumps. To develop a broad-spectrum antiviral drug against the emerging coronavirus infection, this study assessed the antiviral properties of these compounds. The activity of seven types of cardiotonic steroids against the MERS-CoV, SARS-CoV, and SARS-CoV-2 coronavirus varieties was analyzed using immunofluorescence antiviral assay in virus-infected cells. Bufalin, cinobufagin, and telocinobufagin showed high anti-MERS-CoV activities (IC_50_, 0.017~0.027 μM); bufalin showed the most potent anti-SARS-CoV and SARS-CoV-2 activity (IC_50_, 0.016~0.019 μM); cinobufotalin and resibufogenin showed comparatively low anti-coronavirus activity (IC_50_, 0.231~1.612 μM). Differentially expressed genes in Calu3 cells treated with cinobufagin, telocinobufagin, or bufalin, which had high antiviral activity during MERS-CoV infection were analyzed using QuantSeq 3′ mRNA-Seq analysis and data showed similar gene expression patterns. Furthermore, the intraperitoneal administration of 10 mg/kg/day bufalin, cinobufagin, or digitoxin induced 100% death after 1, 2, and 4 days in 5-day repeated dose toxicity studies and it indicated that bufalin had the strongest toxicity. Pharmacokinetic studies suggested that telocinobufagin, which had high anti-coronavirus activity and low toxicity, had better microsomal stability, lower CYP inhibition, and better oral bioavailability than cinobufagin. Therefore, telocinobufagin might be the most promising cardiotonic steroid as a therapeutic for emerging coronavirus infections, including COVID-19.

## 1. Introduction

Severe acute respiratory infectious diseases have been induced by zoonotic beta-coronaviruses such as SARS-CoV, MERS-CoV, and SARS-CoV-2 [1]. Since the outbreak of severe acute respiratory syndrome (SARS) in 2002, highly pathogenic and transmissible coronavirus disease, MERS and COVID-19 have threatened public health and welfare. Even though vaccination against COVID-19 is extensively underway, the number of confirmed cases worldwide is over 200 million with up to 4.3 million deaths till August 2021 [2], indicating that the pandemic is not yet abating. Remdesivir, the first-in-class antiviral drug of choice for COVID-19, has been clinically used for treating COVID-19 patients. However, the development of best-in-class broad-spectrum antivirals which might be able to terminate the current pandemic is still needed.

This study aimed to find candidate natural compounds showing a broad-spectrum antiviral activity against emerging coronavirus infections. This study focused on the antiviral properties of cardiotonic steroids (also known as cardiac glycosides), which are natural compounds with a steroid-like structure. Several cardiotonic steroids, including digoxin, digitoxin, and ouabain, have been reported to inhibit infection by DNA viruses, such as cytomegalo, herpes simplex, and adenovirus, and RNA viruses, such as Ebola, chikungunya, influenza, respiratory syncytial, and human immunodeficiency virus [3,4]. Anti-coronaviral activities of cardiotonic steroids have also been reported in in vitro feline infectious peritonitis virus, human coronavirus OC43 and 229E, MERS-CoV, and SARS-CoV-2 [5,6].

Cardiotonic steroids are named according to their cardiotonic activity. Cardiotonic steroids inhibit the plasma membrane Na^+^/K^+^-ATPase pumps, which increases the intracellular Na^+^ and Ca^+^ levels, decreases intracellular K^+^ levels, and finally increases cardiac contractile force [7]. Cardiotonic steroids such as digitoxin and digoxin have been isolated from *Digitalis lanata* and *D. purpurea.* These compounds are classified as cardenolides and have a steroid ring with a five-carbon unsaturated butyrolactone moiety. Other cardiac steroids such as bufadienolides, including bufalin, cinobufagin, telocinobufagin, bufotalin, cinobufotalin, and resibufogenin, have also been found in Venenum Bufonis, the venom from the skin glands of toad species such as *Bufo bufo gargarizans*. These cardiac steroids have a six-carbon unsaturated pyrone ring attached to the steroid ring [8,9,10]. Approximately 150 bufadienolides have been isolated from Venenum Bufonis that is used as a traditional medicine in East Asia against inflammation and for pain relief, anesthesia, etc. [9,11]. Cardiotonic steroids have become an area of interest due to their bioactive Na^+^/K^+^-ATPase pump inhibition property showing therapeutic potential in various diseases including antitumor cell growth, anti-inflammatory immunomodulation, and antiviral infections [3,7,10,12].

This study aimed to identify an optimal candidate cardiotonic steroid that shows effective broad-spectrum antiviral activity against emerging coronaviruses and high availability for clinical application. Therefore, the anti-coronaviral activity of digitoxin, a type of cardenolide, and bufalin, cinobufagin, telocinobufagin, bufotalin, cinobufotalin, and resibufogenin, all types of bufadienolides, against MERS-CoV, SARS-CoV, and SARS-CoV-2 was analyzed and compared. The differentially expressed genes (DEGs) affected by each compound were investigated, a 5-day repeated dose toxicity study was conducted, and the pharmacokinetics of the selected compounds were explored.

## 2. Materials and Methods

### 2.1. Test Compounds

Digitoxin (PubChem CID 441207), bufalin (PubChem CID 9547215), cinobufagin (PubChem CID 11969542), telocinobufagin (PubChem CID 259991), bufotalin (PubChem CID 12302120), cinobufotalin (PubChem CID 259776), and resibufogenin (PubChem CID 6917974) with ≥98% purity were purchased from ChemFaces Biochemical Co. (Wuhan, China). Compounds were used to make 20-mM stock solutions with dimethyl sulfoxide (Sigma-Aldrich, St. Louis, MO, USA).

### 2.2. Cells and Viruses

Vero (ATCC^®^ CCL-81™) and Calu-3 (ATCC^®^ HTB-55™) cells were purchased from the American Type Culture Collection (Manassas, VA, USA). Vero cells were maintained in Dulbecco’s modified Eagle’s medium (DMEM) (Gibco, Carlsbad, CA, USA), and Calu-3 cells were maintained in Eagle’s minimum essential medium (EMEM, ATCC), both supplemented with 10% fetal bovine serum (FBS, Gibco) and antibiotic–antimycotic solution (Gibco) at 37 °C with 5% CO_2_. MERS-CoV (MERS-CoV/KOR/KNIH/002_05_2015; GenBank accession number KT029139.1) and SARS-CoV-2 (βCoV/KOR/KCDC03/2020) were provided by the Korea Disease Control and Prevention Agency (KDCA). SARS-CoV strain HK39849 was provided by Prof. JSM Peiris from the University of Hong Kong. Virus propagation and plaque assays for titration were performed using Vero cells. Experiments with infectious coronavirus were performed in a biosafety level-3 facility at the Institut Pasteur Korea following the guidelines of the Korea National Institute of Health (KNIH) and using procedures approved by the KDCA.

### 2.3. Immunofluorescence Antiviral Assays

Vero cells (1.2 × 10^4^ cells/384-well black plate) were seeded in DMEM supplemented with 2% FBS and 1X antibiotic–antimycotic solution. After 24 h, the serially diluted compounds and MERS-CoV (0.0625 multiplicity of infection [MOI]), SARS-CoV (0.05 MOI), or SARS-CoV-2 (0.0125 MOI) were added to the plates. At 24 h postinfection (pi), the cells were fixed using 4% paraformaldehyde and stained using the anti-MERS-CoV spike, anti-SARS-CoV spike, or anti-SARS-CoV-2 nucleocapsid antibodies (Sino Biological Inc., Beijing, China); goat anti-rabbit IgG secondary antibody; and Hoechst 33342 (Thermo Fisher Scientific, Waltham, MA, USA). Images were analyzed using the Operetta imaging system (20×; Perkin Elmer Waltham, MA, USA) and Image-Mining 3.0 plug-in software.

### 2.4. Viral Cytopathic Effect Assays

Calu-3 cells (1.5 × 10^4^ cells/384-well white plate) were seeded in EMEM supplemented with 2% FBS and 1X antibiotic–antimycotic solution (Gibco) 24 h prior to the experiment. Serially diluted compounds and 0.004 MOI MERS were added and incubated at 37 °C for 72 h. Cell viability was measured using the CellTiter-Glo^®^ luminescent cell viability assay (Promega Corporation, Madison, WI, USA) according to the manufacturer’s instructions.

### 2.5. RNA Isolation and QuantSeq 3′ mRNA-Seq Analysis

The total RNA of Calu-3 cells infected with or without 0.004 MOI MERS-CoV or treated for 24 h with MERS-CoV, and 10 μM of the indicated compounds was isolated using RNeasy Mini Kits (Qiagen, Valencia, CA, USA). RNA quality was assessed using the Agilent 2100 bioanalyzer with the RNA 6000 Nano Chip (Agilent Technologies, Amstelveen, The Netherlands), and RNA was quantified using an ND-2000 Spectrophotometer (Thermo Fisher Scientific, Waltham, MA, USA). A library was constructed using QuantSeq 3′ mRNA-Seq Library Prep Kits (Lexogen GmbH, Vienna, Austria). High-throughput sequencing was performed as single-end 75 sequencing using NextSeq 500 (Illumina, Inc., San Diego, CA, USA). QuantSeq 3′ mRNA-seq reads were aligned using Bowtie2 [13]. DEGs were determined based on counts from unique and multiple alignments using coverage in BEDTools [14]. The read count (RC) data were processed based on a quantile normalization method using EdgeR within R [15] using Bioconductor [16]. For DEGs, Gene Ontology (GO) analyses [17] were performed using clusterProfiler (Version 3.18.1) [18] in R (Version 4.0.3), which supports the statistical analysis and visualization of functional profiles for genes and gene clusters.

### 2.6. Five-Day Repeated Dose Toxicity Study

Five-week-old male C57BL/6 mice were purchased from Orient Bio (Gyeonggi, Korea). The mice were housed at the Animal Care Facility of the Korea Institute of Toxicology (KIT) under standard laboratory conditions (24 °C, humidity 50%, 12 h day/night cycles) and provided with standard chow diet and drinking water (KIT, Daejeon, Korea). Before the experiments, the mice were acclimated for 7 days. Experimental procedures were approved by the Institutional Animal Care and Use Committee of KIT (approval number, KIT-B118096). Compounds were administrated through intraperitoneal injection at a DMSO:DW:PEG4000 ratio of at 5:55:40 at doses of 2 or 10 mg/kg/day once a day for 5 days. There were five mice in each treatment group. The mice were observed for the duration of drug administration and the body weight was measured daily.

### 2.7. Liver Microsomal Metabolic Stability Assays

Samples of 0.5 mg protein/mL of liver microsomes from mice, rat, or human (Corning, Glendale, AZ, USA) and 1 μM compound were mixed. NADPH-regenerating solution (Corning) was added and incubated at 37 °C for 30 min. The reaction was stopped by the addition of cold acetonitrile and the samples were centrifuged. The supernatant was analyzed using mass spectrometry with high-performance liquid chromatography (HPLC, Agilent).

### 2.8. hERG K^+^ Channel Binding Assays

Predictor hERG fluorescence polarization assay kits (Life Technologies, Carlsbad, CA, USA) were used according to the manufacturer’s instructions. Fluorescence polarization was measured using an Infinite M1000 Pro Microplate Reader (Tecan, Männedorf, Switzerland).

### 2.9. Plasma Protein Binding Assays

Animal plasmas (Innovative Research, Novi, MI, USA) and 5 μM of compounds were incubated in a rapid equilibrium dialysis device system (Thermo Fisher Scientific, Waltham, MA, USA) for 4 h. Cold acetonitrile was added to stop the reaction and the samples were centrifuged. The supernatant was analyzed using mass spectrometry with HPLC (Agilent).

### 2.10. Cytochrome P-450 (CYP450) Enzyme Inhibition Assays

CYP450 enzyme inhibition was tested using P450-gloTM assay kits (Promega Corporation) according to the manufacturer’s instructions. Luminescence was measured using an Infinite M1000 Pro (Tecan) after 20 min of stabilization with a luciferin detection reagent.

### 2.11. Pharmacokinetic Studies

Sprague–Dawley male rats were purchased from NARA-Bio (Pyeongtaek, Korea). The rats were housed under standard laboratory conditions (24 °C, humidity 50%, 12 h day/night cycles), provided with a standard chow diet and drinking water, and allowed to acclimate for 1 week prior to the experiments. All animal procedures were approved by the KRICT Animal Care and Use Committee (approval number, DDP-6500). Doses of 2 mg/kg or 10 mg/kg of compound in 5:55:40 of DMSO:DW:PEG400 were administered intravenously or orally (*n* = 3). Blood samples were collected at indicated time points after drug administration from the retro-orbital venous plexus. After obtaining plasma samples, acetonitrile was added, and the supernatant was collected and analyzed using liquid chromatography–tandem mass spectrometry (LC–MS/MS). Mean plasma concentration–time data were analyzed using noncompartmental methods (Phoenix WinNonlin software, Pharsight Corporation, Mountain View, CA, USA).

### 2.12. Statistical Analysis

Data are presented as the mean ± SEM of at least two independent experiments. Non-linear regression analysis of IC_50_ was conducted using GraphPad Prism^®^ Software V.6.05 for Windows (GraphPad Software Inc., San Diego, CA, USA).

## 3. Results

### 3.1. Anti-MERS-CoV Activity of Cardiotonic Steroids

**Figure 1:**
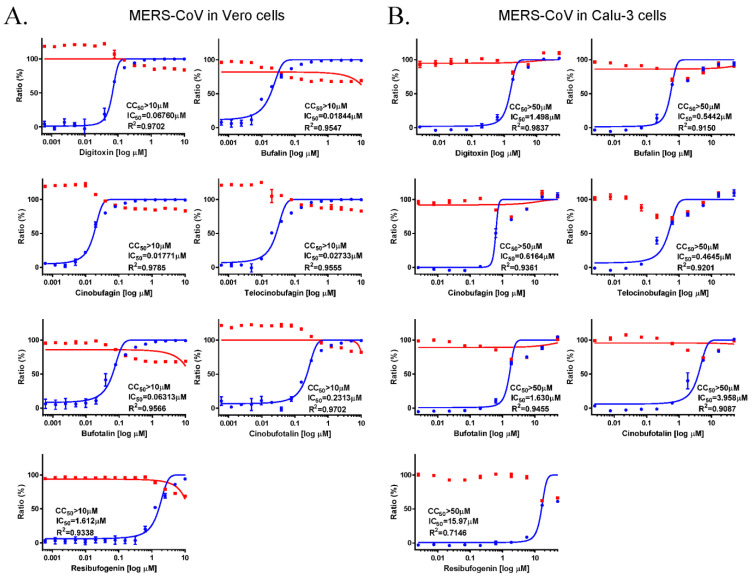
Anti-MERS-CoV activity of the cardiotonic steroids digitoxin, bufalin, cinobufagin, telocinobufagin, bufotalin, cinobufotalin, and resibufogenin. (**A**) The dose–response curve analysis by immunofluorescence staining was performed to determine the antiviral effect of the cardiotonic steroids, ranging between 0.001 and 10 μM in Vero cells. (**B**) The dose–response curve analysis of the viral cytopathic effect was performed to determine the antiviral effects of cardiotonic steroids, ranging between 0.01 and 10 μM in Calu-3 cells. Inhibition of MERS-CoV infection (%; blue circles) and cell viability (%; red squares) were indicated. The IC_50_ values were calculated using non-linear regression analysis. The data represent duplicate experiments and are presented as the mean ± SEM.

The antiviral activity of the cardiotonic steroids, digitoxin, bufalin, cinobufagin, telocinobufagin, bufotalin, cinobufotalin, and resibufogenin were evaluated using immunofluorescence assays in MERS-CoV infected Vero cells. Vero cells were treated with serial dilutions of the compounds during MERS-CoV infection. At 24 h after infection, the Vero cells were stained using the anti-spike protein of the MERS-CoV antibody, and the IC_50_ and CC_50_ of each compound were calculated and compared. The IC_50_ values of each compound were 0.067 μM (digitoxin), 0.018 μM (bufalin), 0.017 μM (cinobufagin), 0.027 μM (telocinobufagin), 0.063 μM (bufotalin), 0.23 μM (cinobufotalin), and 1.612 μM (resibufogenin), with CC_50_ values of >10 μM. Thus, the selectivity indices (SI, CC_50_/IC_50_) were >147.9 for digitoxin, 542.3 for bufalin, 564.7 for cinobufagin, 365.9 for telocinobufagin, 158.4 for bufotalin, 43.2 for cinobufotalin, and 6.2 for resibufogenin (Figure 1A). These data suggested that the strength of the anti-MERS-CoV activity was in the order of cinobufagin ≥ bufalin ≥ telocinobufagin > bufotalin ≥ digitoxin > cinobufotalin > resibufogenin.

The anti-MERS-CoV activity of these compounds in Calu-3 human lung cells, which is the target tissue of coronavirus infection, was examined. The Calu-3 cells were treated with the target compounds and the antiviral activity was determined by evaluating the MERS-CoV virus-induced cytopathic effect (CPE) 3 days after infection. The IC_50_ values were 1.498 μM for digitoxin, 0.544 μM for bufalin, 0.616 μM for cinobufagin, 0.465 μM for telocinobufagin, 1.630 μM for bufotalin, 3.958 μM for cinobufotalin, and 15.970 μM for resibufogenin with a CC_50_ of >50 μM, suggesting SIs of >33.4 for digitoxin, >91.9 for bufalin, >81.1 for cinobufagin, >107.6 for telocinobufagin, >30.7 for bufotalin, >12.6 for cinobufotalin, and >3.1 for resibufogenin (Figure 1B). These data suggested that the anti-MERS-CoV activity in Calu-3 cells was in the order of telocinobufagin ≥ bufalin ≥ cinobufagin > digitoxin ≥ bufotalin > cinobufotalin > resibufogenin. Collectively, these data suggested that all these compounds show potent anti-MERS-CoV activity. Bufalin, cinobufagin, and telocinobufagin had the highest anti-MERS-CoV activity, digitoxin and bufotalin had medium activity, and cinobufotalin and resibufogenin had low activity.

### 3.2. Comparative Gene Expression Analysis of Compound-Treated Calu-3 Cells during MERS-CoV Infection

**Figure 2:**
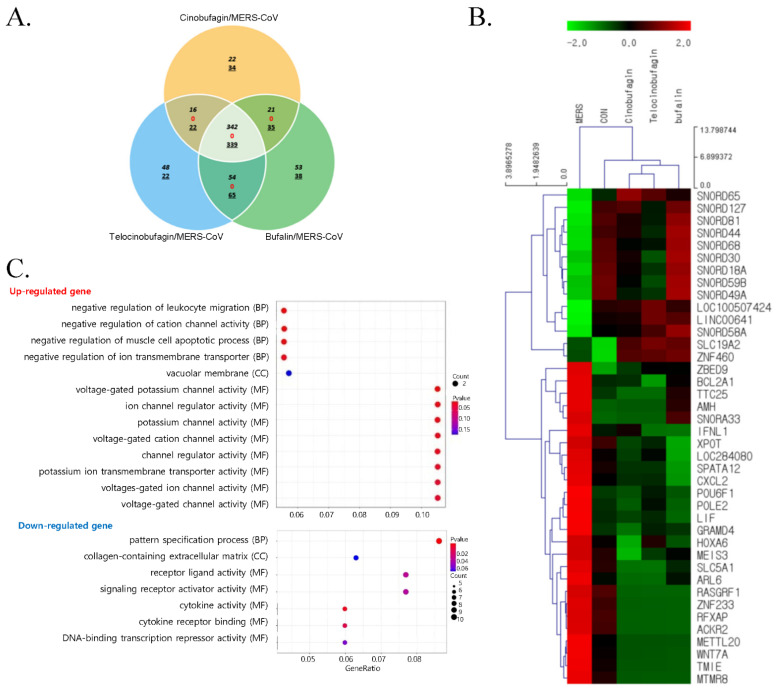
Gene expression patterns following treatment with cinobufagin, telocinobufagin, or bufalin during MERS-CoV infection. (**A**) Venn diagram analysis of the number of overlapping upregulated (in italics), contraregulated (red), or downregulated (underlined) genes by 10 μM cinobufagin, telocinobufagin, or bufalin treatment in Calu-3 cells during 24 h of MERS-CoV infection versus MERS-CoV infection (fold change, 4; log2 normalized RC of ≥8). (**B**) Expression heatmap and clustering analysis of 40 significant differentially expressed genes (DEG) during 24 h of MERS-CoV-infected Calu-3 and control cells treated with cinobufagin, telocinobufagin, or bufalin (fold change, 4; log2 normalized read count [RC] of ≥4). (**C**) Gene Ontology biological processing (BP), cellular component (CC), molecular function (MF) terms of upregulated and downregulated genes in cinobufagin-, telocinobufagin-, or bufalin-treated Calu-3 cells during MERS-CoV infection. The related GO terms were ranked according to gene ratio (selection counts/selection size). The circle size and color indicate the selection counts and *p*-value, respectively (Fold change, 4; log2 normalized RC of ≥2).

To investigate the DEGs in Calu3 cells treated with cinobufagin, telocinobufagin, or bufalin during MERS-CoV infection, QuantSeq 3′ mRNA-seq analysis was performed. When the numbers of commonly expressed genes meeting the criterion of four-fold change (log2 normalized RCs of ≥8) were compared, 342 genes were upregulated and 339 genes were downregulated. There were 1111 genes in the pooled sample (*n* = 3) of Calu-3 cells treated with 10 μM cinobufagin, telocinobufagin, or bufalin during MERS-CoV infection versus MERS-CoV infected cells; there were no contra-regulated genes (Figure 2A). Cluster analysis confirmed the close relationships of the cinobufagin-, telocinobufagin-, or bufalin-treated Calu-3 cells during MERS-CoV infection (Figure 2B). Cells treated with cinobufagin, telocinobufagin, or bufalin during MERS-CoV infection had similar gene expression patterns.

Moreover, 40 significant DEGs were found in Calu-3 cells treated with cinobufagin, telocinobufagin, or bufalin and control cells at 24 h post MERS-CoV infection (Fold change, 4; log2 normalized RC of ≥4) (Figure 2B). Genes related to cell death, BCL2 related protein A1 (*BCL2A1*) and GRAM domain containing 4 (*GRAMD4*), and the immune and inflammatory response related genes, atypical chemokine receptor 2 (*ACKR2*), interferon, lambda 1 (*IFNL1*), C-X-C motif chemokine ligand 2 (*CXCL2*), and leukemia inhibitory factor (*LIF*) were upregulated by MERS-CoV infection, but these genes were downregulated by these compound treatments during MERS-CoV infection. Small nucleolar RNAs, C/D box 18A, 30, 44, 49A, 58A, 59B, 65, 68, 81, and 127 (SNORD18A, SNORD30, SNORD44, SNORD49A, SNORD58A, SNORD59B, SNORD65, SNORD68, SNORD81, and SNORD127, respectively) were downregulated by MERS-CoV infection, but were upregulated when treated with the experimental compounds. In contrast, small nucleolar RNA, H/ACA box 33 (SNORA33) was upregulated by MRES-CoV infection and downregulated by the compounds.

GO analysis of the biological process, cellular component, and molecular function of upregulated genes in the cinobufagin, telocinobufagin, or bufalin treated Calu-3 cells during MERS-CoV infection revealed the enrichment of ion channel activity regulation (Figure 2C). GO analysis of downregulated genes revealed enrichment of biological processes such as pattern specification, and molecular functions such as the activity of receptor and ligands including cytokines.

### 3.3. Anti-SARS-CoV and SARS-CoV-2 Activity of Cardiotonic Steroids

**Figure 3:**
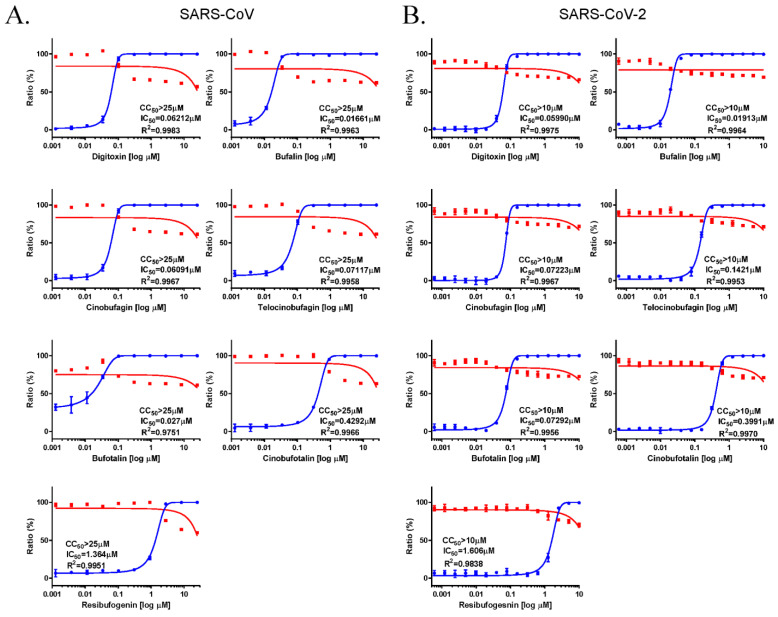
Anti-SARS-CoV and anti-SARS-CoV-2 activity of digitoxin, bufalin, cinobufagin, telocinobufagin, bufotalin, cinobufotalin, and resibufogenin. The dose–response curve analysis using immunofluorescence staining was performed to determine the anti-SARS-CoV (**A**) and anti-SARS-CoV-2 (**B**) activity of cardiotonic steroids in Vero cells (ranging between 0.001 and 10 μM). Inhibition of SARS-CoV and SARS-CoV-2 infection (%, blue circles) and cell viability (%, red squares) were indicated. IC_50_ values were calculated using non-linear regression analysis. The data represent duplicate experiments and are presented as the mean ± SEM.

To examine the broad-spectrum anti-coronavirus activity of the cardiotonic steroids, the antiviral effects of digitoxin, bufalin, cinobufagin, telocinobufagin, bufotalin, cinobufotalin, and resibufogenin against SARS-CoV and SARS-CoV-2 were analyzed using immunofluorescent assays in SARS-CoV and SARS-CoV-2 infected Vero cells. Data from SARS-CoV (Figure 3A) and SARS-CoV-2 (Figure 3B) infections indicated that these compounds had the similar antiviral activity as that against MERS-CoV infection. All of these compounds had effective anti-SARS-CoV and SARS-CoV-2 activity with CC_50_ > 10 μM. Bufalin showed the most potent anti-SARS-CoV (IC_50_ = 0.016 μM) and SARS-CoV-2 (IC_50_ = 0.019 μM) activity. Digitoxin, cinobufagin, telocinobufagin, and bufotalin had similar activity, and cinobufotalin and resibufogenin had comparatively low activity. Overall, these data suggested that these cardiotonic steroids have potent broad-spectrum anti-coronavirus activity.

### 3.4. Toxicity and Pharmacokinetics of Cinobufagin and Telocinobufagin

**Figure 4:**
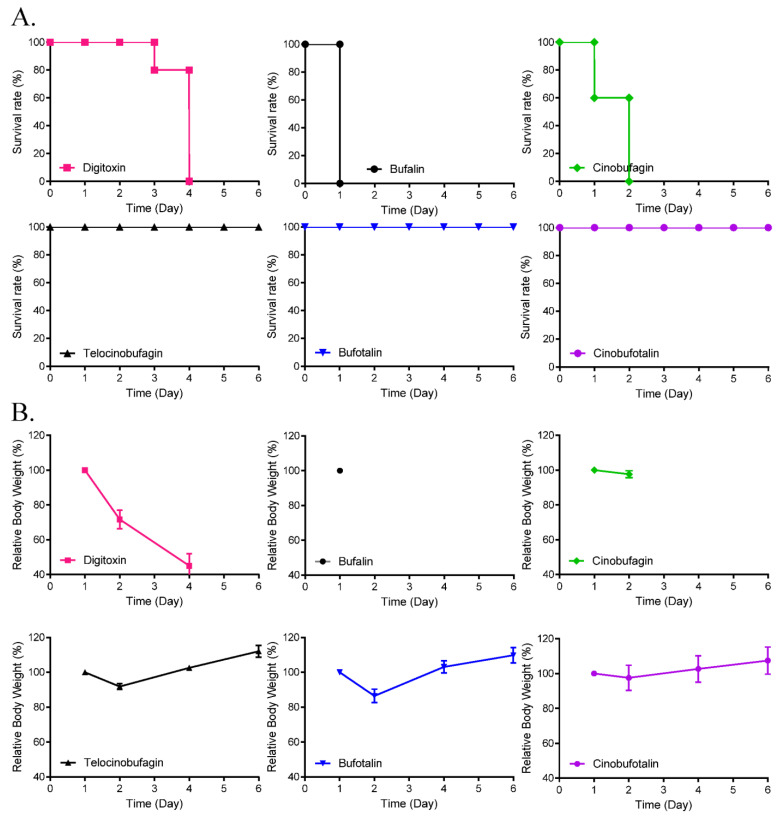
Effects of cardiotonic steroids on 5-day repeated dose toxicity. Survival rate (**A**) and body weight changes (**B**) of mice with intraperitoneal injection of digitoxin, cinobufagin, cinobufotalin, telocinobufagin, bufalin, or bufotalin at a daily dose of 10 mg/kg/day (five mice/group) for 5 days.

To compare the toxicity of the cardiotonic steroids, 5-day repeated dose toxicity studies were performed using all the above-mentioned compounds except resibufogenin, which showed the least antiviral activity. Peritoneal administration of 10 mg/kg/day telocinobufagin, bufotalin, and cinobufotalin for 5 days induced 100% survival. However, the administration of bufalin, cinobufagin, and digitoxin induced 100% death at 1, 2, and 4 days after administration (Figure 4), respectively, although administration of 2 mg/kg/day showed 100% survival (data not shown). These data suggested that bufalin had the strongest toxicity in mice. 

Cinobufagin and telocinobufagin were selected for further investigation and their pharmacological features, including microsomal stabilities (MS), human ether a-go-go (hERG) bindings, plasma protein binding, and CYP450 inhibitions were measured (Table 1). The data from the liver microsomal stability tests showed that cinobufagin was quickly metabolized, with <5% remaining within 30 min, and telocinobufagin remained at 15–30% in mouse, rat, and human, suggesting that telocinobufagin is microsomally more stable than cinobufagin. These compounds interacted with approximately 20% of the hERG channel in hERG channel inhibition assays. The PPB rate of cinobufagin (78–90%) was lower than that of telocinobufagin (96–97%) in mouse and rat. In CYP450 inhibition assays, cinobufagin inhibited 4–46% of isozyme activity, and telocinobufagin inhibited 1.4–21% of activities. The pharmacokinetic properties of cinobufagin and telocinobufagin were analyzed using 1 mg/kg intravenous (IV) and 2 mg/kg oral (PO) injection in male rats. Cinobufagin was not detected in plasma 30 min after PO injection, due to its rapid clearance, and all rats (*n* = 3) died within 10 min following 1 mg/kg IV injection of cinobufagin (data not shown). However, the oral bioavailability of telocinobufagin was 33% (Table 2). Therefore, these data suggested that telocinobufagin was the most acceptable candidate therapeutic drug for COVID-19 among the cardiotonic steroids tested.

## 4. Discussion

**Figure 5:**
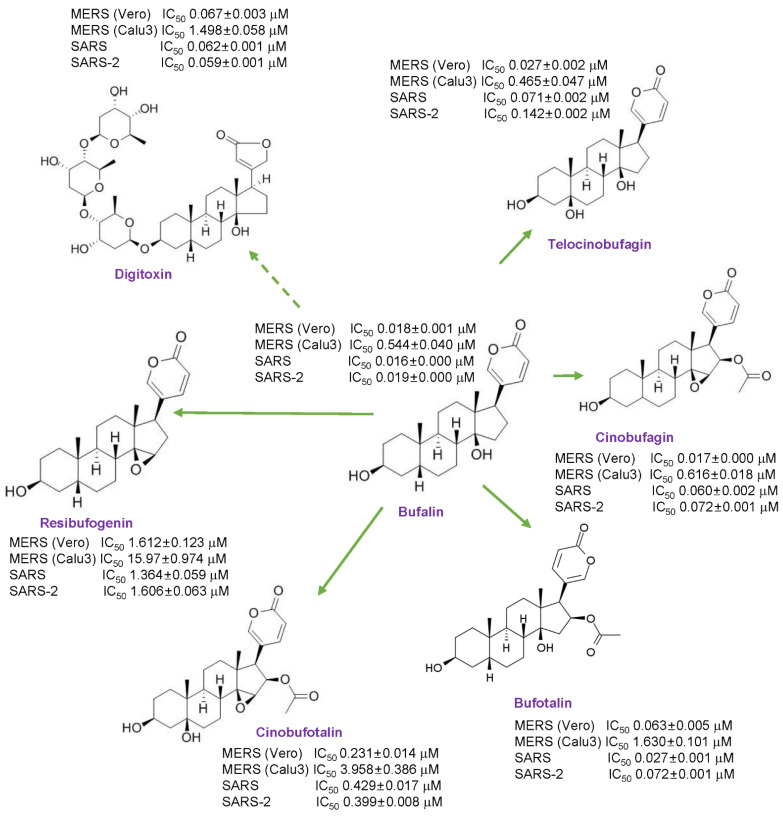
Summary of the anti-coronaviral activity of cardiotonic steroids.

A broad-spectrum anti-MERS-CoV, SARS-CoV, and SARS-CoV-2 activity of the cardiotonic steroids, digitoxin, bufalin, cinobufagin, telocinobufagin, bufotalin, cinobufotalin, and resibufogenin was observed. Among them, bufalin, cinobufagin, and telocinobufagin had high anti-MERS-CoV activity, and bufalin had the most potent anti-SARS-CoV and SARS-CoV-2 activity (Figure 5).

When the DEGs in Calu3 cells treated with the most potent anti-coronaviral compounds, cinobufagin, telocinobufagin, or bufalin, were investigated using QuantSeq 3′ mRNA-seq analysis, similar gene expression patterns were induced by cinobufagin, telocinobufagin or bufalin treatment during MERS-CoV infection. MERS-CoV infection upregulated cell death-related genes and immune- and inflammatory-related genes. However, this upregulated gene expression was reversed by these compounds. Data also suggested that the levels of C/D-class small nucleolar RNAs (SNORDs) and H/ACA small nucleolar RNAs (SNORAs) were regulated by MERS-CoV infection, and these were reversed by these compound treatments. Small nucleolar RNAs (snoRNAs) are noncoding RNAs comprising 67–280 nucleotides in the nucleolus involved in the processing of ribosomal RNA. There are two classes of snoRNAs: SNORDs guiding 2′-*O*-ribose methylation and SNORAs directing pseudouridylation of nucleotides [19,20]. SnoRNAs have a regulatory role in human diseases including neurodegenerative disorders, cancer, and viral diseases [21]. It has been reported that small RNAs are differentially expressed during virus infection, and human SNORA31 variants impaired immunity to herpes simplex virus-1 [22,23]. The snoRNA silencing also reportedly inhibits virus replication [24]. The differentially expressed SNORDs and SNORAs observed during MERS-CoV infection and treatment were consistent with the previous reports. So, further study of the specific mode of actions of differentially expressed snoRNAs is required in more detail whether they mediate the host antiviral response or the virus life cycle. In addition, GO analysis showed that treatment with cinobufagin, telocinobufagin, or bufalin during MERS-CoV infection upregulated the genes involved in the regulation of ion channel activity, and downregulated receptor and receptor ligands including cytokines. Cardiotonic steroids reportedly inhibit the Na^+^/K^+^-ATPase pumps and the inhibition of the ATP1A1α subunit of Na^+^/K^+^-ATPase pumps by bufalin inhibits MERS-CoV infection at an early stage [5]. Due to the inhibition of Na^+^/K^+^-ATPase pumps by cinobufagin, telocinobufagin, or bufalin, the host cells could upregulate the regulation of ion channel activity to compensate for the intracellular ion concentrations and maintain homeostasis. In contrast, MERS-CoV infection induced the production of cytokines such as interferon and activated receptors in Calu-3 cells. However, ligand production and activation induced by MERS-CoV infection were downregulated by cinobufagin, telocinobufagin, or bufalin treatment.

Moreover, the toxicity of digitoxin, bufalin, cinobufagin, telocinobufagin, bufotalin, and cinobufotalin was compared using 5-day repeated dose toxicity studies in mice. Although the intraperitoneal administration of 2 mg/kg/day of these compounds resulted in 100% survival, administration of bufalin, cinobufagin, or digitoxin at 10 mg/kg/day resulted in 100% death at 1, 2, and 4 days after administration, respectively; administration of telocinobufagin, bufotalin, and cinobufotalin at 10 mg/kg/day resulted in 100% survival. These data suggest that bufalin had the highest anti-coronaviral activity as well as the strongest toxicity. Therefore, cinobufagin and telocinobufagin were selected for their high anti-coronavirus activity and low toxicity and the pharmacokinetic properties of these compounds were further examined. These data suggest that telocinobufagin had better microsomal stability and lower CYP inhibition than cinobufagin, although these compounds inhibited hERG channels by approximately 20%, and the PPB rates were >80%. Investigation of the pharmacokinetic properties showed that the oral bioavailability of telocinobufagin was better than that of cinobufagin, suggesting that telocinobufagin was more promising among the cardiotonic steroids for being developed as an anti-coronaviral drug.

## 5. Conclusions

In this study, the anti-coronaviral activity of the cardiotonic steroids, digitoxin, bufalin, cinobufagin, telocinobufagin, bufotalin, cinobufotalin, and resibufogenin against MERS-CoV, SARS-CoV, and SARS-COV-2 was examined and compared. The proof of concept (POC) of cardiotonic steroids was performed only in vitro. Therefore, in vivo POC and the therapeutic target study in detail should be executed in further studies. Investigations into the efficacy of antiviral activity, 5-day repeated dose toxicity, and pharmacokinetic properties suggested that telocinobufagin was the most promising therapeutic candidate among the tested cardiotonic steroids for use against emerging coronaviruses including COVID-19.

## Figures and Tables

**Table 1:** Microsomal stability (MS), hERG inhibition (10 μM), plasma protein binding (PPB), and CYP450 inhibition (10 μM) of cinobufagin (CIN) and telocinobufagin (TEL).

Compound (1 μM)	MS (% of Remaining)			hERG Inhibition (%)	PPB (%)		CYP450 Inhibition (%)				
	Mice	Rat	Human		Mice	Rat	1A2	2C9	2C19	2D6	3A4
CIN	0.18	4.40	2.99	24.6	90.8	78.2	4.09	33.0	46.7	11.0	6.14
	±0.07	±0.51	±0.39	±6.82	±1.74	±8.84	±1.0	±1.2	±9.3	±3.9	±5.3
TEL	21.7	31.9	15.1	22.6	97.8	96.8	6.22	21.1	19.5	1.40	9.42
	±0.90	±1.31	±1.27	±6.02	±0.35	±0.78	±1.3	±2.8	±11.1	±3.2	±5.3

**Table 2:** Rat pharmacokinetic study of telocinobufagin.

Parameters (*n* = 3)	I.V., 1 mg/kg	P.O., 2 mg/kg
Tmax (h)	NA	0.333 ± 0.144
Cmax (μg/h)	NA	0.537 ± 0.227
T_1/2_ (h)	0.225 ± 0.02	0.51 ± 0.407
AUC (μg∙h/mL)	0.604 ± 0.159	0.488 ± 0.204
CL (L/h/kg)	1.73 ± 0.432	not applicable (NA)
V_ss_ (L/kg)	0.275 ± 0.169	NA
F_t_ (%)	NA	33.2

## Data Availability

The data presented in this study are available in this article.
